# Distinct Dengue Disease Epidemiology, Clinical, and Diagnosis Features in Western, Central, and Eastern Regions of Indonesia, 2017–2019

**DOI:** 10.3389/fmed.2020.582235

**Published:** 2020-11-20

**Authors:** R. Tedjo Sasmono, Marsha S. Santoso, Yanuarni W. B. Pamai, Benediktus Yohan, Anna M. Afida, Dionisius Denis, Ingrid A. Hutagalung, Edison Johar, Rahma F. Hayati, Frilasita A. Yudhaputri, Sotianingsih Haryanto, Samuel C. B. Stubbs, Barbara A. Blacklaws, Khin S. A. Myint, Simon D. W. Frost

**Affiliations:** ^1^Eijkman Institute for Molecular Biology, Jakarta, Indonesia; ^2^Santa Elisabeth Hospital, Batam, Indonesia; ^3^Dr. H. M. Ansari Saleh Hospital, Banjarmasin, Indonesia; ^4^Dr. M. Haulussy Hospital, Ambon, Indonesia; ^5^Siloam Hospitals, Jambi, Indonesia; ^6^Department of Veterinary Medicine, University of Cambridge, Cambridge, United Kingdom; ^7^Department of Infectious Disease Epidemiology, London School of Hygiene and Tropical Medicine, London, United Kingdom; ^8^Microsoft Research, Redmond, WA, United States

**Keywords:** arbovirus, dengue, serotypes, chikungunya, clinical, Indonesia

## Abstract

The people of Indonesia have been afflicted by dengue, a mosquito-borne viral disease, for over 5 decades. The country is the world's largest archipelago with diverse geographic, climatic, and demographic conditions that may impact the dynamics of disease transmissions. A dengue epidemiology study was launched by us to compare and understand the dynamics of dengue and other arboviral diseases in three cities representing western, central, and eastern Indonesia, namely, Batam, Banjarmasin, and Ambon, respectively. A total of 732 febrile patients were recruited with dengue-like illness during September 2017–2019 and an analysis of their demographic, clinical, and virological features was performed. The seasonal patterns of dengue-like illness were found to be different in the three regions. Among all patients, 271 (37.0%) were virologically confirmed dengue, while 152 (20.8%) patients were diagnosed with probable dengue, giving a total number of 423 (57.8%) dengue patients. Patients' age and clinical manifestations also differed between cities. Mostly, mild dengue fever was observed in Batam, while more severe cases were prominent in Ambon. While all dengue virus (DENV) serotypes were detected, distinct serotypes dominated in different locations: DENV-1 in Batam and Ambon, and DENV-3 in Banjarmasin. We also assessed the diagnostic features in the study sites, which revealed different patterns of diagnostic agreements, particularly in Ambon. To detect the possibility of infection with other arboviruses, further testing on 461 DENV RT-PCR-negative samples was performed using pan-flavivirus and -alphavirus RT-PCRs; however, only one chikungunya infection was detected in Ambon. A diverse dengue epidemiology in western, central, and eastern Indonesia was observed, which is likely to be influenced by local geographic, climatic, and demographic conditions, as well as differences in the quality of healthcare providers and facilities. Our study adds a new understanding on dengue epidemiology in Indonesia.

## Introduction

Indonesia is the world's largest archipelagic country of about 18,000 islands spread over 1,904,569 km^2^, which can be roughly divided into three regions: western, central, and eastern. The tropical climate of this country is favorable for the transmission of mosquito-borne diseases, including dengue and other arboviral diseases (1). This condition also has the potential for concurrent infections of multiple pathogens, which may have severe clinical and epidemiological implications (2). Outbreaks of dengue virus (DENV) and chikungunya virus (CHIKV) are common, while Zika virus (ZIKV) infection was reported as early as 1978 in Central Java and was recently detected in Sumatra (3–5).

Indonesia is one of the countries with the highest dengue burden globally (6). In 2016, the national incidence rate (IR) of dengue disease in Indonesia was 78.9 per 100,000 population, which rose from 50.8 per 100,000 population in 2015 (7). While dengue surveillance data in Indonesia is now accumulating, data on CHIKV and ZIKV transmission is very limited. Furthermore, their infection rates tend to be underestimated in part because of their clinical similarity with dengue. Misdiagnosis is also common. In addition, given the large number of cases with an unidentified etiology, there may be other pathogens in circulation that remain unknown or undetected.

Dengue is a systemic viral infection caused by DENV with a global burden of an estimated 50 million infections annually around the world (8). Dengue cases have been reported in all 34 provinces of Indonesia, with all four DENV serotypes (DENV-1,−2,−3, and−4) reported to be circulating in the country (9). The clinical manifestations of dengue can be classified based on severity into dengue fever (DF), dengue hemorrhagic fever (DHF), and dengue shock syndrome (DSS) (10). While dengue severity and transmissibility have been correlated with viral genetics (11), all four of the serotypes of DENV can cause severe and fatal disease, although DENV-2 and DENV-3 have been more associated with severe disease (12, 13).

With the vastness of the archipelago, a wide range of geographic, climatic, and demographic conditions as well as inequalities in infrastructure and socioeconomic development are evident in Indonesia. The western regions of the country tend to be more densely populated and more developed in terms of infrastructure, in contrast to the eastern counterparts (14). Moreover, inequality in the availability of health-related infrastructure and access to services has become a problem. Provinces in western regions of Indonesia tend to have higher overall Public Health Development Index scores compared to eastern regions (15). Whether the epidemiological characteristics of dengue and other arboviral diseases are influenced by these diverse conditions is unknown.

**Figure 1:**
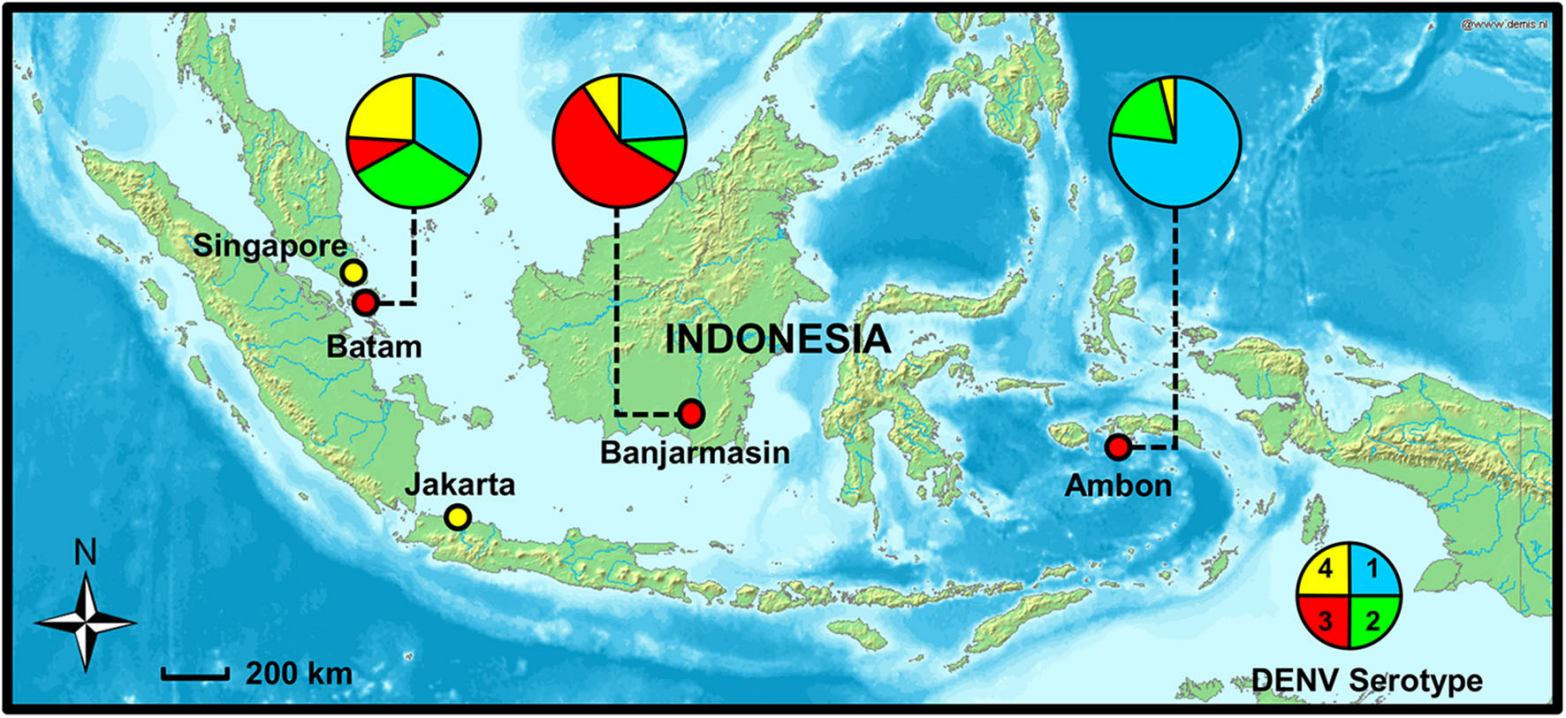
Study sites in Batam, Banjarmasin, and Ambon cities with dengue virus (DENV) serotype distribution (shown in pie charts), 2017–2019. Map source: http://www2.demis.nl/worldmap/mapper.asp.

This study aims to determine and compare the clinical, demographic, and virological features of dengue in different regions of Indonesia. Three study sites representing the western, central, and eastern regions of Indonesia were chosen: Batam in Riau Island province, Banjarmasin in South Kalimantan province, and Ambon in Maluku province, respectively (Figure 1). The dengue IRs in these provinces are dynamic, and continued transmission of dengue is evident (7). The study also aims to determine dengue epidemiology in areas with high (Batam and Banjarmasin) and low (Ambon) dengue IRs, within the same period. Febrile patients presenting to hospitals with symptoms similar to dengue were recruited and examined for dengue and other arboviral infections.

## Materials and Methods

### Ethical Clearance

The study protocol was reviewed and approved by the Eijkman Institute Research Ethics Committee (EIREC) with approval No. 113/2017. Written informed consent was obtained from patients recruited for the study. Consent from parents or legal guardians were obtained on behalf of minors.

### Study Sites and Dengue Incidences

The study participants were all patients presenting dengue-like symptoms and attending a designated referral hospital in each city where the study was conducted. Patients were recruited from Santa Elisabeth Hospital in Batam (the largest city in Riau Island, 1°05′N 104°02′E), Dr. H. M. Ansari Saleh Hospital in Banjarmasin (the capital city of South Kalimantan, 3°20′S 114°35′E), and Dr. M. Haulussy Hospital in Ambon (the capital city of Maluku, 3°42′S 128°10′E) during September 2017–2019. The study period covered two typical dengue peak seasons in the area. Batam's rainy season usually occurs from October to April with an average annual temperature of 26.8°C, while Banjarmasin occurs from November to April with an average annual temperature of 26.7°C, and Ambon from May to September each year with an average annual temperature of 26.5°C (https://en.climate-data.org). The average humidity in Batam, Banjarmasin, and Ambon are 81, 80, and 84%, respectively (www.timeanddate.com). In 2016, the population of Batam, Banjarmasin, and Ambon were 1,236,399, 675,440, and 427,934, respectively (Statistics Indonesia, 2016. Available at www.bps.go.id).

In 2016, dengue IRs in Riau Island, South Kalimantan, and Maluku provinces were 64.1, 101.1, and 21.2 per 100,000 population, respectively. The data showed an increase from the previous year, which were 51.4, 91.9, and 4.6 per 100,000 population, respectively (7).

### Patient Recruitment, Sample Collection, and Laboratory Examinations

Sample size for patient recruitment was calculated using the population prevalence estimation formula with an expected prevalence of 25%, confidence level of 95%, and precision of 5%. To obtain sufficient representation, ~300 patients were required for Batam and Banjarmasin. For Ambon, due to the relatively low IR of dengue compared to the other two cities, sample size calculation was further corrected using the finite population formula, requiring ~100 patients. Children and adults between the age of 1 and 65 years, with fever over 38°C for <5 days and symptoms suggestive of dengue disease were recruited by research teams on each site. Those with clear symptoms of upper respiratory or gastrointestinal tract infections and unwilling to participate in this study were excluded. Upon hospital admission, single 3- to 5-ml blood samples were taken during the acute phase. Sera were separated by centrifugation and kept frozen until further processing. The demographics and basic hematology data (hemoglobin, hematocrit, erythrocyte, platelet, and leucocyte count) were obtained using standard questionnaire and from hospital medical records.

### Case Definitions

Dengue-like illness was defined as acute fever reported by the patients (16), and in our study, with additional clinical assessment on symptoms, which were similar to dengue and the patients presenting to hospitals during dengue season. Probable and confirmed dengue was defined based on the WHO-SEARO's 2011 guidelines (10). Probable dengue are cases of acute fever accompanied by at least one of the clinical signs of dengue, such as malaise, arthralgia, rash, and retro-orbital pain, as well as a single positive result in IgM or IgG (10). These cases were confirmed for dengue when there was at least one of the following laboratory results: (1) isolation of dengue virus, (2) detection of DENV NS1 antigen, (3) detection of DENV genomic sequence by RT-PCR, and (4) a four-fold increase in IgG or IgM DENV-specific antibodies (10). The latter was irrelevant for our study as only single acute samples were collected. Cases were diagnosed as non-dengue if they tested negative for DENV NS1, RT-PCR, and IgM.

In terms of severity, patients were classified into dengue fever (DF) if they reported clinical symptoms of dengue but without signs of plasma leakage (10). Patients exhibiting signs of plasma leakage were classified into DHF, which is shown by any of the following signs: a rise in hematocrit of ≥20% from baseline, pleural effusion, ascites, or hypoalbuminemia (10). Patients were categorized into DSS if they showed signs of shock, which include tachycardia, delayed capillary refill time, cold extremities, poor peripheral pulse, hypotension, and pulse pressure ≤20 mmHg (10).

### DENV NS1 Antigen Detection and Serological Tests

Collected serum samples were tested for the presence of DENV NS1 antigen and anti-DENV IgG and IgM antibodies using Standard Q Dengue Duo (Biosensor, Korea) rapid-tests on sites, performed in accordance with manufacturer's instructions. Patients who tested positive for dengue IgM/IgG with clinical symptoms of dengue were classified as “probable dengue,” while patients who tested positive for the dengue NS1 antigen and/or RT-PCR were categorized as “confirmed dengue” (10). To assess the diagnostic agreement and accuracy in clinical settings between study sites, the sensitivities, specificities, and predictive values of NS1 and IgM/IgG diagnostics were compared with RT-PCR results as the gold standard. These parameters were also stratified by age, gender, fever day onset, and serotype to compare the rapid-tests' performance in different conditions.

### DENV RNA Extraction, RT-PCR Detection and Serotyping

Serum samples were transported to Eijkman Institute for Molecular Biology in Jakarta under appropriate cold chain maintenance for further tests to confirm dengue and/or other arbovirus infections. Viral RNA was extracted from 200 μl of sera using MagNA Pure LC Total NA extraction kit (Roche, Mannheim, Germany) in the MagNA Pure LC 2.0 extraction system (Roche). DENV nucleic acid detection was performed using Simplexa® Dengue qRT-PCR (DiaSorin, Saluggia, Italy) to simultaneously detect and serotype DENV as previously described (17). All samples for all three study sites underwent the same protocols.

### Other Arbovirus Detection

All samples that were negative for DENV NS1 and RT-PCR (*N* = 461) were subjected to other arbovirus screening using broadly reactive Alphavirus (18) and Flavivirus (19) group-specific RT-PCRs. Virus genetic material was amplified on 25 μl of OneStep RT-PCR kit (Qiagen, Hilden, Germany) reaction, containing 400 μM dNTPs, 1 μM primers, and 5 μl of template RNA. The thermal cycling conditions were reverse transcription for 30 min at 50°C, PCR activation for 15 min at 95°C, followed by 35 cycles of amplification at 94°C for 45 s, 50°C for 1 min, and 72°C for 1 min, and final extension at 72°C for 10 min. Reactions were run on 1% agarose gels, and DNA fragments were excised and purified using QIAquick gel extraction kit in accordance with the manufacturer's instruction (QIAGEN). DNA sequencing was performed using BigDye™ Terminator v3.1 cycle sequencing kit, following the manufacturer's protocol (Applied Biosystems, USA) on a 3500XL genetic analyzer (Applied Biosystems, USA). The resulting DNA sequences were compared to banked specimens using NCBI BLAST (https://blast.ncbi.nlm.nih.gov/Blast.cgi).

### Statistical Analysis

Categorical data between cities were compared using Pearson's Chi-square or Fisher's exact-tests as appropriate, while age and hematological data between cities were compared using Kruskal–Wallis-tests. Regression analyses were performed using binomial logistic regression of clinically relevant potential covariates such as age and gender for reported symptoms. The performance of DENV NS1 and IgG/IgM rapid-tests were evaluated using *Z*-tests for proportions. Statistical analysis was performed using R Studio software (http://www.r-project.org) with a *p*-value of <0.05 considered as statistically significant.

## Results

### Patient Characteristics and Clinical Features

**Figure 2:**
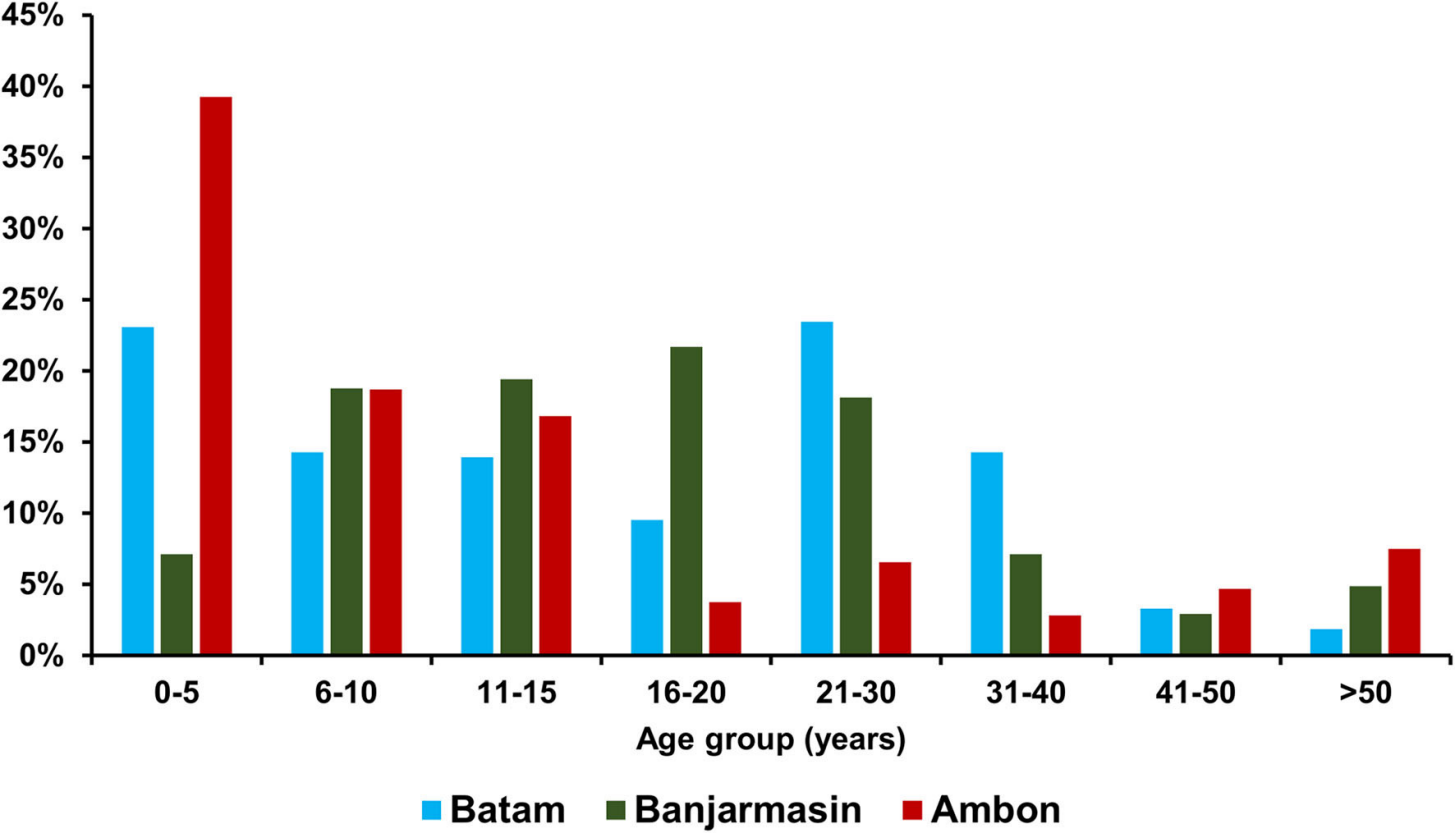
Age distribution of dengue-like illness patients in Batam, Banjarmasin, and Ambon.

Of the recruited 732 dengue-like illness patients across three study sites during the same period, 315 were from Batam, 310 from Banjarmasin, and 107 from Ambon. The median age of patients in Ambon was significantly younger [9 years old (y.o.), interquartile range (IQR) = 4–16 y.o.] than those in Batam (15 y.o, IQR = 6–26 y.o.) and Banjarmasin (16 y.o, IQR = 10–23 y.o.) (*p* < 0.001). Detailed age distribution is shown in Figure 2. In terms of gender, more male patients were observed in Batam and Ambon with a female-to-male ratio of 1:1.1 and 1:1.5, respectively. However, no statistically significant differences were observed between the groups. Equal female-to-male ratio was observed in Banjarmasin.

**Figure 3:**
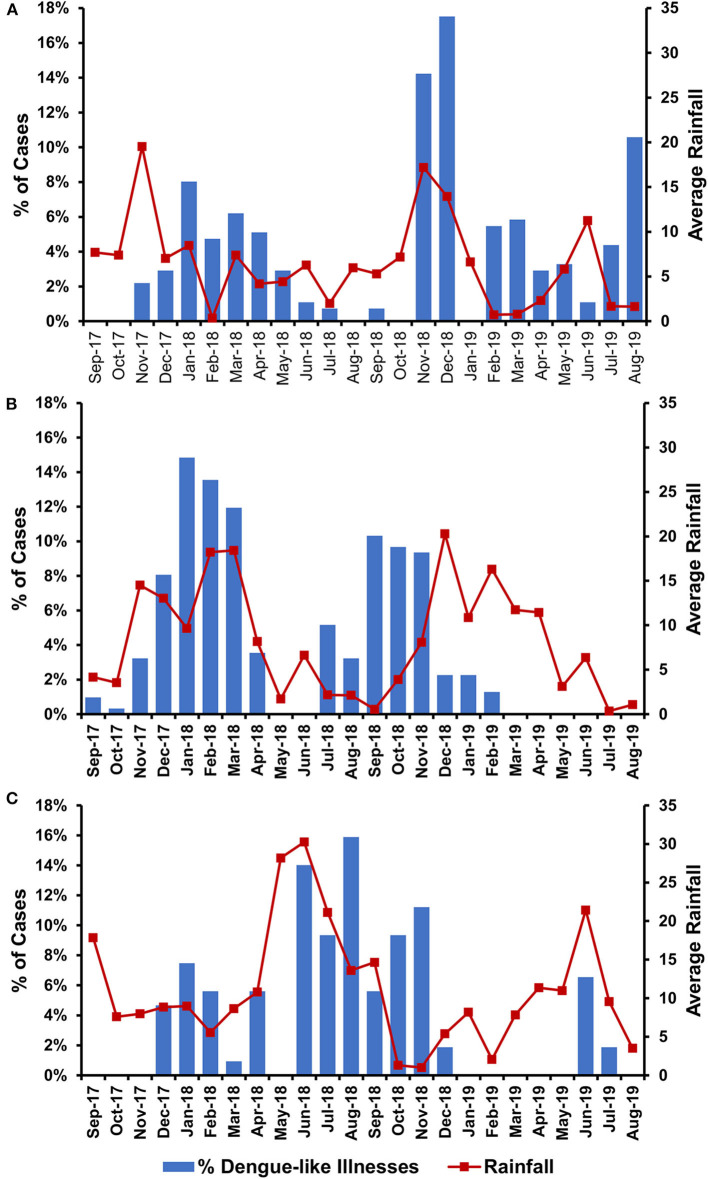
Temporal distribution of dengue-like illness incidences in relation with average monthly rainfall in Batam **(A)**, Banjarmasin **(B)**, and Ambon **(C)**.

To understand the temporal distribution of dengue-like illness incidence in all study sites, we analyzed hospital admission dates and correlated them with climatic data. We observed that the temporal distribution of cases was generally similar between study sites, except for Ambon. Cases in Batam and Banjarmasin follow seasonal patterns, which peaked during December–May each year, while in Ambon, during June–November. In general, the temporal distribution of dengue-like illness cases in all three study sites peaked around the occurrence of monsoon season in each region (Figure 3). Dengue-like illness patients in the three sites exhibited all dengue clinical spectrum (Table 1). Patients in Batam tend to report less dengue symptoms compared to those in Banjarmasin and Ambon. The most reported symptoms in Banjarmasin were loss of appetite, malaise, and nausea, while in Ambon were headache, stomachache, retro-orbital pain, and malaise. Latent class analysis showed that febrile dengue-suspected cases in Ambon reported significantly more headache, myalgia, nausea, loss of appetite, malaise, and stomachache compared to cases in Banjarmasin and Batam (*p* < 0.001), though these classes are not associated with DENV detection and are more related to site.

**Table 1:** Clinical symptoms of dengue-like illness patients in Batam, Banjarmasin, and Ambon.

**Symptoms**	**Proportion of reported symptoms**, ***N*** (%)^*^			*p*-value^**^
	**Batam (*N* = 315)**	**Banjarmasin (*N* = 310)**	**Ambon (*N* = 107)**	
Headache	185 (58.9)^a^	193 (64.8)^a^	106 (99.1)^b^	<0.001
Retro-orbital Pain	36 (11.5)^a^	46 (16.3)^a^	102 (95.3)^b^	<0.001
Myalgia	43 (13.7)^a^	136 (46.9)^b^	101 (94.4)^c^	<0.001
Arthralgia	32 (10.2)^a^	75 (26.4)^b^	81 (75.7)^c^	<0.001
Nausea	101 (32.2)^a^	225 (73.5)^b^	101 (94.4)^c^	<0.001
Loss of appetite	145 (46.2)^a^	269 (87.6)^b^	98 (91.6)^b^	<0.001
Rash	23 (7.3)^a^	84 (29.6)^b^	50 (46.7)^c^	<0.001
Bleeding^***^	7 (2.2)^a^	117 (38.2)^b^	16 (15.1)^c^	<0.001
Enlarged liver	0 (0.0)^a^	19 (6.8)^b^	10 (9.3)^c^	<0.001
Fluid accumulation	2 (0.6)^a^	4 (1.4)^a^	30 (28.0)^b^	<0.001
Stomachache	42 (13.4)^a^	178 (58.6)^b^	102 (96.2)^c^	<0.001
Malaise	154 (49.0)^a^	246 (80.9)^b^	102 (95.3)^c^	<0.001
Anxiousness	53 (16.9)^a^	73 (25.4)^b^	81 (75.7)^c^	<0.001
Drowsiness	68 (21.7)^a^	64 (21.9)^a^	66 (62.3)^b^	<0.001
Allergy	18 (5.7)^a^	1 (0.3)^b^	0 (0.0)	<0.001

* *Cells in a row with the same superscript letter are ones whose difference in proportions are not significantly different from each other with a Bonferroni-adjusted p-value threshold of 0.05*.

** *Pearson's Chi-Square-Test*.

*** *Bleeding manifestations include any of the following: petechiae, purpura, ecchymosis, epistaxis, gum bleeding, gastrointestinal bleeding, hemoglobinuria/hematuria, and hypermenorrhea*.

### Dengue and Other Arbovirus Confirmatory Diagnoses

To determine whether the dengue-like illness patients were indeed infected by DENV, we tested the patients' sera using DENV NS1 antigen rapid diagnostic-test (RDT) during the first day of hospital admission. In addition to NS1 antigen detection, we also conducted real-time RT-PCR-tests to simultaneously detect and serotype the infecting DENV in patients' sera. Both NS1 antigen and nucleic acid detection are considered as confirmatory dengue diagnosis (10). Among 732 patients, 271 (37.0%) of them were virologically confirmed dengue (Table 2). The highest percentage of virologically confirmed dengue patients was observed in Banjarmasin (42.6%) and was lowest in Ambon (30.8%) (Table 2). In addition to the virologically confirmed dengue, there were 152 (20.7%) patients that were categorized as probable dengue based on their positivity in IgM and/or IgG antibodies (Table 2). Even with all dengue confirmatory tests, there were still 294 (40.2%) of dengue-like illness patients that remained negative (Table 2).

**Table 2:** Dengue and other arbovirus laboratory diagnoses on acute-patient serum samples.

**Category**	**City**			**Total**
	**Batam (*N* = 315)**	**Banjarmasin (*N* = 310)**	**Ambon (*N* = 107)**	
**Dengue virus (DENV) antigen and RNA detection**, ***N*** **(%)**				
NS1-positive, RT-PCR-negative	6 (1.9)	30 (14.3)	6 (5.6)	42 (5.7)
NS1-negative, RT-PCR-positive	37 (11.7)	32 (10.3)	19 (17.8)	88 (12.0)
NS1- and RT-PCR-positive	63 (20.0)	70 (22.6)	8 (7.5)	141 (19.3)
**Virologically confirmed dengue**	**106 (33.7)**	**132 (42.6)**	**33 (30.8)**	**271 (37.0)**
**DENV serotype distribution**, ***N*** **(%)**				
DENV-1	34 (34)	23 (22.6)	20 (74.1)	77 (33.6)
DENV-2	33 (33)	9 (8.8)	5 (18.5)	47 (20.5)
DENV-3	9 (9)	56 (54.9)	0 (0.0)	65 (28.4)
DENV-4	24 (24)	9 (8.8)	1 (3.7)	34 (14.9)
Mixed serotypes	0 (0)	5 (4.9)	1 (3.7)	6 (2.6)
**DENV serological diagnosis**, ***N*** **(%)**				
IgM-positive, IgG-negative	21 (6.7)	31 (10.3)	2 (1.9)	55 (7.5)
IgG-positive, IgM-negative	10 (3.2)	28 (9.0)	12 (11.2)	50 (6.8)
IgM- and IgG-positive	17 (5.4)	153 (49.4)	17 (15.9)	187 (25.5)
**Probable dengue**	**17 (5.4)**	**116 (37.4)**	**19 (17.8)**	**152 (20.7)**
**Non-dengue patients (all negative)**	**191 (60.6)**	**48 (15.5)**	**55 (51.4)**	**294 (40.2)**
**Other arboviruses detection of dengue-NS1- and RT-PCR-negative samples (*****N*** **=** **461)**				
Pan-flavivirus (dengue) RT-PCR-positive	8	9	3	20
Pan-flavivirus (non-dengue) RT-PCR-positive	0	0	0	0
Pan-alphavirus RT-PCR-positive	0	0	1	1

*Bold indicates the significant p-values*.

RT-PCR was used to determine the serotypes of infecting DENV. While all four serotypes can be detected, distinct serotype predominance was observed; DENV-1 and−2 in Batam, DENV-3 in Banjarmasin, and DENV-1 in Ambon (Figure 1 and Table 2). However, a fluctuation of serotype predominance pattern was found, particularly in Batam, in which DENV-2 prevalence in late 2017 to 2018 was followed by DENV-4 predominance in 2019 (Supplementary Figure 1). We also detected mixed infections among the patients in Banjarmasin (four patients infected with DENV-1 and DENV-3, and one patient infected with DENV-1 and DENV-4) and Ambon (one patient infected with DENV-1 and DENV-3). On the whole, the predominant DENV serotypes circulating in Indonesia during the study was DENV-1 (33.6%), followed by DENV-3 (28.4%), DENV-2 (20.5%), and DENV-4 (14.9%).

A subset of samples with DENV NS1- and RT-PCR-negative results (*N* = 461, 63.0%) were also subjected to a second round of RT-PCRs to detect possible infection with other arboviruses. The pan-flavivirus RT-PCR was positive for 20 samples in this subset, with DNA sequencing of PCR amplicons confirming DENV (Table 2). These DENV-positive samples were serotyped as seven DENV-2 and one DENV-4 in Batam; three DENV-2, five DENV-3, and one DENV-4 in Banjarmasin; and three DENV-2 in Ambon (Table 2). Except DENV, no other flaviviruses were detected.

The pan-alphavirus RT-PCR only detected one positive sample. DNA sequencing was performed on the PCR amplicon, and the resulting sequence was matched with the chikungunya virus genome. The chikungunya patient was a 3-year-old female from Ambon. She was admitted to the hospital at day 2 of fever and reported to experience symptoms of fever, headache, retro-orbital pain, myalgia, arthralgia, nausea, loss of appetite, rash, mucosa bleeding, anxiousness, drowsiness, and allergy. She was provisionally diagnosed as dengue. However, her basic hematology results were normal, with hematocrit at 39.2%, platelet count of 198,000/μl, and leukocyte counts of 8,400/μl. Dengue NS1 rapid-test and DENV RT-PCR detection were negative. Dengue IgM RDT was negative, while the IgG RDT was positive.

### Characteristics of Virologically Confirmed Dengue Patients

Dengue-confirmed patients in Ambon were significantly more likely to be younger (median 5 y.o., range 1–52 y.o.) compared to those in Banjarmasin (median 16 y.o., range 1–51 y.o.) and Batam (median 17 y.o., range 1–58 y.o.) (*p* < 0.001). A higher percentage of dengue cases in Banjarmasin had positive IgG results using rapid-tests (72.6%) compared to dengue cases in Ambon (55.8%) and Batam (22.0%), implying that Banjarmasin may have more secondary cases compared to the other two cities. A large proportion of dengue cases in Batam manifested as the less severe DF (84.9%), while in Banjarmasin, there was an almost equal proportion of DF and DHF cases, and in Ambon, there were more DHF (60.6%) cases compared to DF cases (Table 3).

**Table 3:** Severity, immunology, and hematology features of virologically confirmed dengue (*N* = 271) cases in Batam, Banjarmasin, and Ambon.

**Parameters**	**City**			***p*-value**
	**Batam**	**Banjarmasin**	**Ambon**	
**Severity**, ***N*** **(%)**				
Dengue fever (DF)	90 (84.9)	67 (50.8)	13 (39.4)	** <0.001^a^^,^^c^**
Dengue hemorrhagic fever (DHF)	16 (15.1)	65 (49.2)	20 (60.6)	
**Hematology features, median (range)**				
Platelet (×10^3^/μl)	118.5 (75.8–160.8)	51.5 (34.5–80.0)	77 (43.0–145.0)	** <0.001^b^^,^^d^**
Hematocrit (%)	40.1 (36.6–43.2)	41.3 (38.85–44.45)	36.8 (32.5–41.5)	** <0.001^b^^,^^e^**
WBC (×10^3^/μl)	3.3 (2.5–4.6)	3.1 (2.4–4.5)	5.9 (3.7–10.2)	** <0.001^b^^,^^f^**

a *Pearson's chi square-test*.

b *Kruskal–Wallis-test*.

c *Post-hoc Bonferroni: p < 0.001 (Batam–others), p < 0.001 (Banjarmasin–others), p = 0.017001 (Ambon–others)*.

d *Post-hoc Dunn-test: p < 0.001 (Ambon–Banjarmasin), p = 0.0301 (Ambon–Batam), p = 0.0268 (Banjarmasin–Batam)*.

e *Post-hoc Dunn-test: p < 0.001 (Ambon–Banjarmasin), p < 0.001 (Ambon–Batam), p < 0.001 (Banjarmasin–Batam)*.

f *Post-hoc Dunn-test: p < 0.001 (Ambon–Banjarmasin), p < 0.001 (Ambon–Batam), p < 0.001 (Banjarmasin–Batam)*.

*Bold indicates the significant p-values*.

DHF cases were more likely to have thrombocytopenia, elevated hematocrit, and leucopenia, but this was only statistically significant for platelet counts and hematocrit (Table 4). There were no DSS cases or deaths in our study.

**Table 4:** Hematology features of virologically confirmed dengue (*N* = 271) cases in Batam, Banjarmasin, and Ambon by severity.

**Median (range)**	**Severity**		***p*-value**
	**DF**	**DHF**	
Platelet (×10^3^/μl)	100.5 (75.3–149.5)	33.0 (18.0–45.0)	** <0.001**
Hematocrit (%)	39.9 (36.5–42.7)	42.3 (38.6–45.5)	**0.008**
WBC (×10^3^/μl)	3.3 (2.4–4.7)	3.4 (2.7–4.9)	0.670

*Bold indicates the significant *p*-values*.

The logistic regression analyses on covariates potentially affecting the symptom variables showed that age and gender did not add significantly to the model. In terms of the different DENV serotypes, there was no significant correlation observed between the reported symptoms and any particular serotype (Table 5).

**Table 5:** DENV clinical symptoms and serotype relationships.

**Variables**	**Proportion of reported symptoms**, ***N*** (%)^*^				***p*-value**
	**DENV-1 (*N* = 77)**	**DENV-2 (*N* = 47)**	**DENV-3 (*N* = 65)**	**DENV-4 (*N* = 34)**	
Headache	51	34	44	22	0.906
Retro-orbital Pain	25	10	15	6	0.645
Myalgia	32	15	31	10	0.879
Arthralgia	18	10	20	5	0.645
Nausea	47	26	47	11	0.291
Loss of appetite	58	29	54	19	0.731
Rash	23	14	27	4	0.164
Bleeding	14	5	24	2	0.568
Enlarged liver	2	2	1	0	0.992
Fluid accumulation	5	2	1	0	0.967
Stomachache	36	17	33	7	0.546
Malaise	55	34	51	16	0.190
Anxiousness	24	11	11	8	0.705
Drowsiness	28	16	18	6	0.495
Allergy	2	5	2	0	0.220

* *Binomial logistic regression analysis*.

### Dengue Diagnosis Agreement Among Study Sites

To compare the diagnostic performance and capability of each study site in diagnosing dengue among all cases, we assessed the agreement between NS1 RDT and RT-PCR data. Combining all data from three study sites, we observed that the overall sensitivity and specificity of the NS1 RDT were 61.6 and 91.6%, respectively. There was no significant difference in overall sensitivity and specificity of NS1-tests alone between fever onset of less and over 3 days. However, combining the NS1 with IgM results significantly improved overall sensitivity from 61.6 to 78.2% (*p* < 0.001), but significantly lowered the specificity from 91.6 to 68.7% (*p* < 0.001).

Differences in diagnostic performance parameters were observed between the three cities. Rapid diagnostic-test performed in samples collected in Ambon had significantly lower sensitivity compared to those in Batam and Banjarmasin. In contrast, samples collected in Batam had significantly higher specificity compared to those in Banjarmasin and Ambon. The trend of increased sensitivity and lowered specificity when NS1 was combined with IgM was also observed when samples were stratified into the city groups, although statistical significance was only observed in Banjarmasin (Table 6).

**Table 6:** Sensitivity and specificity of dengue rapid diagnostic tests in clinical setting.

**Parameter**	**Sensitivity (95% CI)**			***p*-value**
	**Batam**	**Banjarmasin**	**Ambon**	
NS1 only^a^^,^^b^	63.0 (52.7–72.3)	68.6 (58.6–77.3)	29.6 (14.5–50.3)	** <0.001**
IgM only^c^^,^^d^	27.3 (19.0–37.3)	67.0 (56.5–76.2)	25.9 (11.9–46.6)	** <0.001**
NS1 + IgM^e^^,^^f^	70.7 (60.6–79.2)	94.7 (87.5–98.0)	48.1 (29.2–67.7)	** <0.001**
*p*-value of NS1 only vs. combined NS1 and/or IgM	0.3154	** <0.001**	0.2642	
**Parameter**	**Specificity (95% CI)**			***p*****-value**
	**Batam**	**Banjarmasin**	**Ambon**	
NS1 only^a^^,^^b^	97.2 (93.7–98.9)	85.6 (79. 9–89.9)	92.5 (83.8–96.9)	** <0.001**
IgM only^c^^,^^d^	94.9 (90.7–97.3)	38.1 (31. 2–45.5)	85.0 (74.9–91.7)	** <0.001**
NS1 + IgM^e^^,^^f^	92.5 (87.9–95.5)	36.0 (29.2–43.3)	82.5 (72.0–89.8)	** <0.001**
*p*-value of NS1 only vs. combined NS1 and/or IgM	**0.049**	** <0.001**	0.09426	

a *Post-hoc sensitivity: p = 1 (Batam–others), p = 0.20157 (Banjarmasin–others), p = 0.0018618 (Ambon–others)*.

b *Post-hoc specificity: p < 0.001 (Batam–others), p < 0.001 (Banjarmasin–others), p = 1 (Ambon–others)*.

c *Post-hoc sensitivity: p < 0.001 (Batam–others), p < 0.001 (Banjarmasin–others), p = 0.20502 (Ambon–others)*.

d *Post-hoc specificity: p < 0.001 (Batam–others), p < 0.001 (Banjarmasin–others), p = 0.011796 (Ambon–others)*.

e *Post-hoc sensitivity: p = 0.7071 (Batam–others), p < 0.001 (Banjarmasin–others), p < 0.001 (Ambon–others)*.

f *Post-hoc specificity: p < 0.001 (Batam–others), p < 0.001 (Banjarmasin–others), p = 0.016554 (Ambon–others)*.

*Bold indicates the significant p-values*.

The sensitivity of the NS1 antigen rapid-test was also significantly different between serotypes, with DENV-1 cases having a significantly lower sensitivity (42.9%, *p* < 0.001) compared to other serotypes, and DENV-2 cases having significantly higher sensitivity (89.4%, *p* < 0.001) compared to other serotypes (Table 7).

**Table 7:** Performance of DENV NS1 antigen RDT against four different serotypes of DENV.

**Serotype**	**Sensitivity (95% CI)**	***p*-value**
DENV-1 (*N* = 77)	42.9 (31.81–54.63)	** <0.001^a^**
DENV-2 (*N* = 47)	89.4 (76.11–96.02)	
DENV-3 (*N* = 65)	70.8 (58.00–81.06)	
DENV-4 (*N* = 34)	47.1 (30.16–64.60)	

a *Post-hoc Bonferroni result: p < 0.001 (DENV-1 vs. others), p < 0.001 (DENV-2 vs. others), p = 0.091 (DENV-3 vs. others), p = 0.095 (DENV-4 vs. others)*.

*Bold indicates the significant p-values*.

## Discussion

This study reports the demographic, clinical, virological, serology, and diagnostic features of dengue and other arboviral infection in three cities representing three different regions in Indonesia. The vast geographical area of Indonesia warrants a comprehensive study to understand the epidemiology of dengue and other arboviral diseases, which was conducted in parallel to provide a comprehensive dengue epidemiology data in Indonesia.

Temporal data indicated that the peak number of dengue-like illness cases in our study coincided with months of heavy rainfall in the areas (Figure 2). This is generally in accordance with dengue seasonal patterns in Indonesia, in which epidemics are usually associated with increased rainfall (20). Of note is the dengue peak season in Ambon, which occurred during June–November, different from the common dengue peak seasons reported in Indonesia, which tends to occur during the first 6 months of the year (21, 22). However, dengue incidence was observed to peak in Ambon following the local annual rainy season. Nevertheless, to our knowledge, our data is the first to report the temporal difference of dengue peak seasons in Indonesia, particularly in the eastern region.

Among dengue-like illness patients recruited in this study, 37.0% were virologically confirmed for dengue, and 20.7% were probable dengue. Altogether, the data showed that the burden of dengue in Indonesia is still high. Also noted is that different cities show different proportions of dengue disease, which may be caused by the diverse climatic and demographic conditions including herd immunity. We noticed that a relatively low number of dengue patients were admitted to the hospital in Ambon compared to those in Batam and Banjarmasin. This is only to be expected since Ambon (Maluku province) is one of the provinces with low dengue IR (7).

In all three study sites, the majority of confirmed dengue cases occurred in children and adolescents under the age of 20 years, similar to previous studies conducted in Southeast Asia (23) as well as other areas in Indonesia such as Palembang (24), Semarang (25), and Jayapura (26), which are located in the western, central, and eastern regions of the country, respectively. In general, demographic characteristics such as age and gender of the patients did not significantly contribute to the presence or absence of symptoms. However, certain symptoms were found to have a significant geographical variation, which is not associated with the different DENV positivity rates between cities. This may be related to the differences in reporting styles between different health facilities, which may be due to cultural differences of how patients in different cities report their symptoms, or differences in diligence of staff in different hospitals in recording patient symptoms. The lower Public Health Development Index in Eastern regions of Indonesia (15) may be associated with healthcare systems with less experience and facilities, which may cause more severe dengue cases in Ambon. Furthermore, although DENV serotypes have been associated with clinical symptoms (13, 27), we did not observe a significant relationship between infecting DENV serotype and clinical symptoms.

Compared to Ambon and Batam, dengue cases in Banjarmasin were more likely to be secondary infections and showed more severe manifestations in terms of hematology data and reported symptoms, including bleeding manifestations. This is in line with previous studies that observed that secondary dengue infections lead to more severe disease than primary infections through antibody-dependent enhancement (ADE) mechanisms (28, 29). The possibility of higher proportion of secondary infection in Banjarmasin may be attributed to the dengue outbreak in the city in the preceding year, i.e., in 2015, in which dengue IR in the province was ranked 5th in Indonesia, a drastic increase from 25th in 2014 (30), with IR increasing further in 2016 (7). Differences in immunologic status reflect the diversity in dengue herd immunity across Indonesia. Previous model-based studies showed that the herd immunity threshold required to block viral transmission is ~50–85% (31, 32), leading to the possibility of future outbreaks in areas with low dengue herd immunity.

The dynamics of dengue epidemiology in the three study sites was also evident in terms of DENV serotype distribution. Although all four serotypes were circulating, the predominance of DENV serotype was different between cities. Almost equal proportion of DENV-1 and DENV-2 were predominant in Batam, while DENV-3 was the most prevalent serotype in Banjarmasin, and DENV-1 in Ambon. This data shows that the distribution of DENV serotype in Indonesia could not be generalized across the whole country at any given period. Spatiotemporal dynamics of serotype distribution is also supported by previous studies (9, 22). The DENV-3 predominance in Banjarmasin is similar to nearby cities, namely, Samarinda and Balikpapan, South Kalimantan in 2015–2016 (33). The DENV-1 predominance in Ambon is of particular interest, especially whether this serotype is directly related with the low dengue cases in the city. Viral genetic analysis is currently underway to fully understand the molecular evolution and the genetic diversity of DENV in the area. Altogether, our study was the first to report the DENV serotype distributions in Batam, Banjarmasin, and Ambon.

The differential diagnoses of dengue include other arboviral diseases (10) such as chikungunya, Japanese encephalitis (JE), and Zika viruses. In this study we also screened the dengue-negative samples using pan-flavivirus and pan-alphavirus panels. West Nile (34) and Zika viruses (4) were previously detected among sera tested negative for DENV using the same approach. While ZIKV and JEV have been serologically detected in most of Indonesian provinces (5, 35), our study failed to detect other flaviviruses.

Our study shows that pan-flavivirus one-step RT-PCR^19^ with 95–100% sensitivity developed over 2 decades ago, is still a good screening assay since it could capture dengue cases tested negative by the commercial RT-PCR assay used in this study. This also suggests that although commercial dengue RT-PCR detection kits are available and widely used, there are possibilities that not all DENV strains can be detected probably due to PCR mispriming. High mutational rates of DENV and genetic diversity have been reported to influence the sensitivity of RT-PCR (17).

The screening of 461 dengue-negative samples using pan-alphavirus only detected one chikungunya patient in Ambon. Chikungunya is reported to be endemic in Indonesia (36), and with clinical similarity with dengue, misdiagnosis is common as with the Ambon case. Cocirculation of CHIKV with DENV has been reported in Indonesia, but at higher rates (37, 38). The documented circulation of chikungunya in Ambon in the 1970s to the 1980s (36), with the single chikungunya case detected in our study may reflect the endemicity of this arbovirus in Ambon.

Rapid-tests for dengue are a fast and convenient method for diagnosing dengue infections in the clinical setting. Accessibility and availability of advanced diagnostic procedures are not distributed evenly across the diverse regions of Indonesia. We compared the performance of dengue RDT conducted in the field to the “gold-standard” of real-time RT-PCR. The overall sensitivity and specificity of the NS1 RDT in our study samples were 61.6 and 91.6%, respectively, which is comparable to previously published studies in Asia and South America (39, 40). In our study, sensitivity for dengue diagnosis was improved when the NS1 result was read together with IgM, which is included in the RDT kit and thus convenient to use concurrently with the NS1-test. This finding is similar to a study conducted in Sri Lanka, which found improved levels of sensitivity and specificity when NS1 rapid-test was combined with IgM (41). However, in our study, we found that while sensitivity improved with combined results, specificity levels decreased. These changes in sensitivity and specificity were only significant for samples with an onset of fever of 3 days and shorter, which is unexpected since IgM usually rises after 3 days of fever. It may be an underestimation by the patients as data on days of fever onset in our study was collected through self-reporting with questionnaires.

In terms of diagnostic sensitivity in detecting DENV serotypes, we observed that the NS1 RDT used in this study was less sensitive against DENV-1 (42.9%) and DENV-4 (47.1%). This highlights the importance of continuous evaluation and development of DENV NS1 rapid-tests. The lower sensitivity of NS1 diagnostic against DENV-4 was reported previously in Indonesia (42, 43), but not for DENV-1, which is in line with other countries in Asia (39, 44). This is in contrast with findings from Latin America where the sensitivity of NS1 rapid-tests was highest in DENV-1 infections (40). The differences in local DENV genotypes in different geographic locations may underline the varying sensitivity of NS1 diagnostics. As observed in our study, Ambon showed the lowest NS1 sensitivity compared to other cities, most likely due to a predominance in DENV-1 infection in that region. Given the diverse clinical, virological, and immunological conditions across the sites, the differences in dengue RDT performance when used in different regions in Indonesia should be anticipated.

While this study provides new information on dengue epidemiology in Indonesia, limitations exist. Our study did not assess the entomological aspect of the dengue epidemiology; therefore, we cannot determine the contribution of vector control on dengue incidences in the study sites. We also did not thoroughly analyze weather data such as humidity and temperature. Nevertheless, we believe that we have shed some light on the influence of local weather conditions on dengue incidence.

## Conclusion

This study reveals that the dengue epidemiology can be dynamic across the Indonesian archipelago even during the same period. The diverse geographic, climatic, demographic, and population characteristics in different regions of Indonesia most likely contribute to the varied clinical, immunological, and virological features. Furthermore, the DENV serotype distribution is distinct between cities and cannot be generalized for the whole country. This information adds further understanding of dengue epidemiology in Indonesia and may aid in better dengue diagnosis and clinical management in the country.

## Data Availability Statement

The raw data supporting the conclusions of this article will be made available by the authors, without undue reservation.

## Ethics Statement

The studies involving human participants were reviewed and approved by Eijkman Institute Research Ethics Committee. Written informed consent to participate in this study was provided by the participants or participants' legal guardian/next of kin.

## Author Contributions

RS conceptualized the study, wrote, reviewed, and edited the original draft, was in charge of the visualization, supervision, project administration, and funding acquisition. MS was responsible for the formal analysis, investigation, writing, reviewing, and editing the article. YP, AA and IH was in charge of the resources and data curation. BY and SS took part in the formal analysis, investigation, writing, reviewing, and editing the article. DD took part in the formal analysis and investigation. IH was responsible for the resources and data curation. EJ, RH, and FY were in charge of the formal analysis and investigation. SH was responsible for the resources. BB was in charge of the formal analysis and writing, reviewing, and editing of the article. KM was responsible for the conceptualization, supervision, writing, reviewing, and editing of the article. SDWF took part in the conceptualization, formal analysis, writing, reviewing, editing of the article, supervision, project administration, and funding acquisition. All authors contributed to the article and approved the submitted version.

## Conflict of Interest

SDWF was employed by Microsoft Research. The remaining authors declare that the research was conducted in the absence of any commercial or financial relationships that could be construed as a potential conflict of interest.

## Supplementary Material

Supplementary Figure 1 Monthly DENV serotype distribution in Batam **(A)**, Banjarmasin **(B)**, and Ambon **(C)**.
