# Supplementary figures and images for: Distinct Dengue Disease Epidemiology, Clinical, and Diagnosis Features in Western, Central, and Eastern Regions of Indonesia, 2017–2019
Source: Front Med (Lausanne). 2020 Nov 20;7:582235. doi: 10.3389/fmed.2020.582235 (PMC7737558; doi:10.3389/fmed.2020.582235)

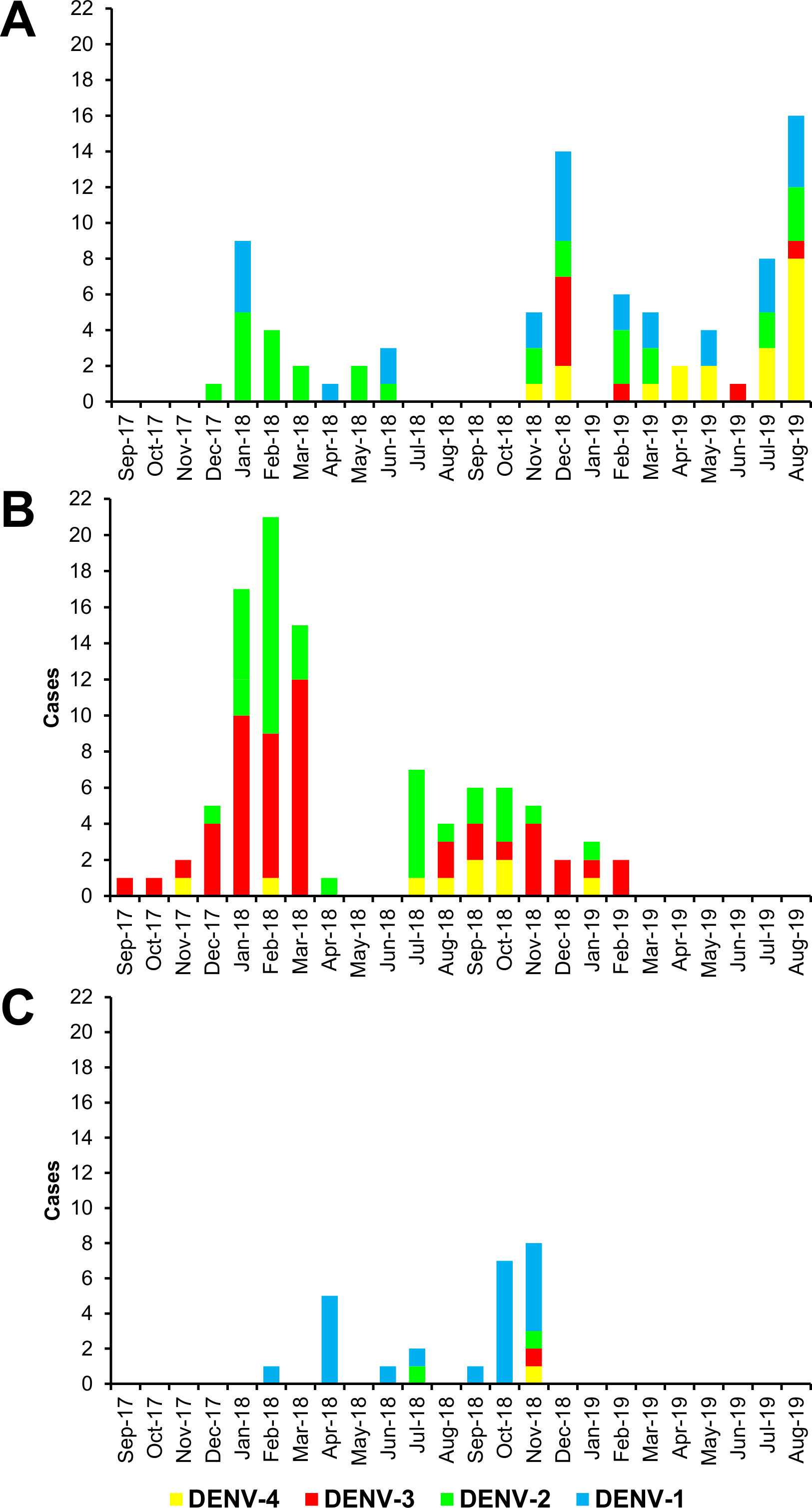

Supplement: Supplementary Figure 1 — Monthly DENV serotype distribution in Batam (A), Banjarmasin (B), and Ambon (C). [file Image_1.JPEG]
